# Upregulation of miR-1254 promotes Hepatocellular Carcinoma Cell Proliferation, Migration, and Invasion via Inactivation of the Hippo-YAP signaling pathway by decreasing PAX5

**DOI:** 10.7150/jca.49680

**Published:** 2021-01-01

**Authors:** Xu Lu, Chao Yang, Yuanchang Hu, Jian Xu, Chengyu Shi, Jianhua Rao, Weixin Yu, Feng Cheng

**Affiliations:** 1 Hepatobiliary Center, The First Affiliated Hospital of Nanjing Medical University; Key Laboratory of Liver Transplantation, Chinese Academy of Medical Sciences, NHC Key Laboratory of Living Donor Liver Transplantation; Nanjing 210029, Jiangsu Province, China.; 2 Department of General Surgery, Changzhou Jintan District People's Hospital; Changzhou 213200, Jiangsu Province, China.

**Keywords:** miR-1254, PAX5, Hippo signaling pathway, Progression, Metastasis, HCC

## Abstract

Increasing evidence suggests that microRNAs (miRNAs) affect the progression of hepatocellular carcinoma (HCC). However, the exact function and mechanism of miR-1254 in HCC remains unclear. This study explored the effects of miR-1254 on the biological behavior of HCC cells and determined the underlying mechanism. RT-qPCR was used to detect the expression of miR-1254. Gain- or loss-of-function assays determined if miR-1254 affected the biological function of HCC cells *in vitro*. Dual luciferase reporter assays confirmed the target gene of miR-1254. Tumor xenografts in mice were used to explore the effects of miR-1254 on tumorigenesis and metastasis of HCC. miR-1254 was upregulated in HCC tissues and cell lines and linked to larger tumor size, aggressive vascular invasion and higher Edmondson grade. Lentiviral-based overexpression and knockdown experiments indicated that miR-1254 promoted proliferation, migration, invasion, and the epithelial-mesenchymal transition of HCC cells. The paired box gene 5 (PAX5) was downregulated in HCC tissues, negatively correlated with miR-1254 expression, and confirmed to be a direct target of miR-1254. Restoration of PAX5 reversed the effects of miR-1254 on the biological behavior of HCC cells. Advanced mechanism studies suggested that PAX5 might mediate miR-1254 by regulating the Hippo signaling pathway. Tumor xenografts in mice confirmed that miR-1254 promoted tumorigenesis and metastasis, and led to poor survival. In conclusion, miR-1254 promoted proliferation, migration, and invasion of HCC cells via decreasing Hippo signaling through targeting PAX5 *in vitro* and *in vivo*. This miRNA might be a therapeutic target for HCC.

## Introduction

Hepatocellular carcinoma (HCC) is one of the most frequent malignancies with the sixth highest incidence and fourth highest mortality in the world 1. Due to the prevalence of hepatitis B virus, patients with HCC are more common in China 2. With surgical resection and transcatheter arterial chemoembolization, the clinical outcomes of HCC have been gradually improving. However, recurrence and metastasis are still problems for long-term survival of patients with HCC 3. Thus, investigating the development and progression of HCC is essential for early diagnosis and treatment.

MicroRNAs (miRNAs) are a class of evolutionary conserved, small, noncoding RNAs that consist of 18-25 nucleotides. Research shows that miRNAs bind to 3'-untranslated regions (3'-UTRs) to downregulate the expression of target mRNAs, leading to degradation or suppression of mRNA translation 4. In addition, previous studies suggest that miRNAs also bind to the 5'-UTRs of mRNAs 5. Intriguingly, an early study indicates that miR-10a binds to the 5'-UTR of ribosomal protein mRNAs and enhances their translation 6. Abundant studies support that miRNAs affect progression of cancers, including proliferation, invasion, apoptosis and migration of tumor cells 7. Recently, miR-1254 was found to regulate tumorigenesis in several cancers such as non-small cell lung carcinoma, and cervical, breast, gastric, and colorectal cancer 8-12. However, the precise function of miR-1254 in HCC remains unclear. In this study, we explored the effects of miR-1254 on the biological behavior of HCC cells and further determined the underlying mechanism.

The paired box (PAX) genes are a family of transcription factors. They regulate differentiation, migration, and proliferation of cells 13. The family is divided into nine members according to structural similarity. Some (PAX3, PAX4, PAX6, PAX7) contain a full homeodomain, some (PAX2, PAX5, PAX8) contain a partial homeodomain and the others (PAX1 and PAX9) contain none at all 14. The paired box gene 5 (PAX5) is extensively studied in lymphoma and lymphocytic leukemia. It acts as an oncogene related to developmental defects of B cells 15. However, PAX5 is mostly regarded as a tumor suppressor in non-lymphoid cancers. Studies suggest that PAX5 mediates a p53 pathway to repress tumorigenesis in gastric cancer 16. In breast cancer, studies demonstrate that PAX5 suppresses breast cancer progression by upregulating miR-215 17. Moreover, PAX5 was found to inhibit non-small cell lung cancer cell proliferation and metastasis by downregulating the β-catenin pathway 18. However, no reports have investigated the association between miR-1254 and PAX5 in HCC.

Numerous studies confirm that signaling pathways are essential in tumorigenesis in human cancers 19-22. Research supports that the Hippo signaling pathway is important in inhibition of tissue overgrowth and tumorigenesis 23. The dysregulation of the Hippo pathway affects human cancers such as prostate, liver, colorectal, and gastric cancer 24-27. The Hippo pathway includes a large tumor suppressor (LATS) kinase that causes the phosphorylation of yes-associated protein (YAP). This process results in more p-YAP retained in cytoplasm, inhibiting transcription of genes that increase cell proliferation and decrease apoptosis 28. However, correlations between the miR-1254/PAX5 axis and Hippo signaling pathway and their effects on HCC cell biological behavior remain unknown.

Our study showed that miR-1254 facilitated HCC proliferation, migration, invasion, and metastasis and functioned as a biomarker for prognosis of HCC. We also determined that miR-1254 negatively regulated PAX5 through decreasing Hippo signaling. This newly discovered miR-1254/PAX5/Hippo signaling pathway may be essential in HCC progression and metastasis. Thus, knowledge of this pathway may assist with finding a novel strategy for treating HCC.

## Material and Methods

### Patients and tissues

Human HCC tissues and paired adjacent normal tissues were obtained from 50 patients with HCC who underwent liver resection at the Hepatobiliary Center, The First Affiliated Hospital of Nanjing Medical University (NMU), China. After resection, all tissues were rapidly frozen in liquid nitrogen. Pathological diagnoses were carried out by the Department of Pathology, which confirmed that all tumor samples were HCC. Our study was approved by the Ethics Committee of the First Affiliated Hospital of NMU. Before liver resection, all patients signed a written informed consent form and agreed to tissue collection for scientific use. The demographic and pathological information of patients is in Table 1.

### Cell culture

The human HCC cell lines Hep3B, MHCC97-H, and Huh-7, and the human normal liver cell line LO2 were from the Chinese Academy of Sciences (Shanghai, China). All cells were cultured in Dulbecco's Modified Eagle's Medium (DMEM; Life Technologies, Carlsbad, CA, USA) supplemented with 10% fetal bovine serum (Life Technologies) and antibiotics (100 U/mL penicillin G and 100 mg/mL streptomycin) at 37°C in a humidified incubator with 5% CO_2_ atmosphere.

### Real-time quantitative polymerase chain reaction (RT-qPCR)

Total RNA was extracted from tissues and cells with TRIzol (Invitrogen, USA). Reverse transcription was with Prime Script RT reagent kits (Takara, China). SYBR Green Master (Takara) was used for quantitative PCR. Primer for PAX5 was from Realgene (Nanjing, China). Primers for miR-1254 and U6 were from RiboBio (Guangzhou, China). Primer sequences were PAX5 forward: 5′-ACTTGCTCATCAAGGTGTCAG-3′, PAX5 reverse: 5′-TCCTCCAATTACCCCAGGCTT-3′, β-actin forward: 5′-TGACGTGGACATCCGCAAAG-3′, β-actin reverse: 5′- CTGGAAGGTGGACAGCGAGG-3′. We used U6 as the control for miR-1254, β-actin as the control for PAX5, and the 2^-ΔΔCT^ method to calculate relative expression levels in samples.

### Fluorescence *in situ* hybridization (FISH)

Expression of miR-1254 in HCC tissues and paired adjacent normal tissues was measured by FISH. From miRBase (www.mirbase.org), we acquired the human miR-1254 sequence 5′-AGCCUGGAAGCUGGAGCCUGCAGU-3′. Locked nucleic acid-based probes against the mature miRNA sequence were used. The 5′-FAM-labeled miR-1254 probe sequence was 3′-TCGGACCTTCGACCTCGGACGTCA-5′. Probe was from Service Bio (Wuhan, China).

### Establishment of stably transfected cells

LV3-hsa-miR-1254-pre-microRNA vector (pre-miR-1254), LV3-hsa-miR-1254-sponge inhibitor vector (miR-1254-inhibitor or anti-miR-1254), vector containing the PAX5 DNA sequence (lv-PAX5), lentiviral vector containing PAX5 siRNA hairpin sequence (PAX5-shRNA) and the respective negative control (NC) vector were designed and constructed by GenePharma (Shanghai, China). After infecting HCC cells with lentiviruses, we used 7 μg/mL puromycin (Sigma-Aldrich, USA) to select cells that were transfected successfully.

### Cell counting kit-8 (CCK-8) assays

Cell Counting Kit-8 (CCK-8) (Dojindo, Japan) was used to detect cell proliferation according to the manufacturer's instructions. Cells were seeded in 96-well plates (2 × 10^3^ cells/well) with 100 μl 10% serum-containing DMEM in wells for 6 days. At a fixed time of day, CCK8 reagent was added to wells and cells incubated for 2 h at 37°C. Absorbance was analyzed spectrophotometrically at 450 nm to evaluate cell proliferation.

### Ethynyl-2'-deoxyuridine (EdU) proliferation assays

EdU proliferation assays (RiboBio, China) were carried out to measure cell proliferation. Cells were seeded in 96-well plates (2 × 10^3^ cells/well) with 100 μL 10% serum-containing DMEM per well for 24 h. Cells were incubated with 50 μM EdU in serum-free DMEM for 2 h at 37°C, followed by fixing in 4% formaldehyde for 30 min on the second day. Glycine was used to neutralize formaldehyde. After permeabilizing with 0.5% TritonX-100 for 10 min at room temperature, 1×Apollo reaction cocktail (100 μl) was added to wells for 30 min. Nuclei were stained with 1×DAPI (100 μL). Cells were imaged under a fluorescence microscope (Nikon, Japan).

### Soft agar growth assays

Anchorage-independent growth of tumor cells was estimated by soft agar growth assays. First, 1 × 10^4^ transfected HCC cells were suspended using DMEM with 0.7% agar. Cells were plated on the top of a layer of 1.4% medium agar. Dishes were marked and incubated at 37°C for 10 days. We counted and photographed viable colonies (≥ 0.1 mm).

### Cell migration and invasion assays

We used Transwell chambers (Millipore, USA) to test the migration and invasion ability of cells. For migration assays, cells were cultured with serum-free DMEM in the upper chamber, and the lower chamber was filled with 10% serum-containing DMEM. For invasion assays, cells were seeded in the upper chamber with a bottom coated with Matrigel membrane. After incubating for 24 hours, 0.1% Crystal Violet was used to stain cells that migrated or invaded across the Transwell membrane for 30 min. Experiments were performed in triplicate.

### Wound-healing assays

Stably transfected HCC cells were seeded in 6-well plates and grown to 95%-100% confluence overnight. A 200-μl sterile pipette tip was used to scratch cells to create a linear wound. Wells were washed with PBS twice to remove suspended cells. Cells were cultured in serum-free DMEM. Wound recovery was observed after 0 and 48 h. At the same position under a microscope, the distance between wound sides was imaged and measured. Experiments were performed in triplicate.

### Western blotting

Total proteins were extracted with RIPA lysis buffer (Beyotime, Shanghai, China), and electrophoresed using 10% sodium dodecyl sulfate polyacrylamide gel electrophoresis and transferred to polyvinylidene fluoride membranes (Bio-Rad, Hercules, CA, USA). Membranes were blocked in Quick Block (Beyotime, Shanghai, China) for 15 min, then incubated with specific primary antibodies (1:1000) at 4°C overnight. Anti-PAX5 (Biorbyt, Cambridge, UK), anti-slug, anti-E-cadherin, anti-vimentin, anti-p-LATS1, anti-LATS1, anti-p-YAP, and anti-YAP (Cell Signaling Technology, USA) were used. Membranes were incubated in secondary antibodies at room temperature for 1 hour. TBST was used to wash unbound antibodies. An enhanced chemiluminescence detection system was used to detect target proteins. GAPDH and β-actin were internal controls.

### Immunofluorescence analysis

First, 4% paraformaldehyde was used to fix transfected cells and 0.3% Triton X-100 was used to permeabilize the cells. Then immunofluorescent staining with anti-E-cadherin, anti-vimentin, and anti-YAP (Cell Signaling Technology, USA) was applied to the cells for 16 h. Finally, the cells were washed and incubated with fluorescence-labeled antibody for 30 min.

### Immunohistochemistry (IHC)

HCC tissues and paired adjacent normal tissues were fixed in 4% formalin and embedded using paraffin. After blocking, paraffin was cut into 4-μm sections and incubated with primary antibodies for PAX5 (Biorbyt, Cambridge, UK) and Ki-67 (Cell Signaling Technology,USA) at 4°C overnight. After washing with PBS, slices were incubated in secondary antibody at 37°C for 1 h and stained with 3,3-diaminobenzidine solution for 3 min. Nuclei were counterstained with hematoxylin.

### Dual luciferase reporter assays

The wild-type and mutant-type sequences of PAX5-3′-UTR were synthesized by GenePharma (Shanghai, China), and inserted into the pmiR-GLO dual-luciferase vector. Wild-type (WT) PAX5-3′-UTR and mutant-type (MUT) PAX5-3′-UTR were used to transfect cells that overexpressed miR-1254 or its control using Lipofectamine 2000 (Invitrogen, USA). Renilla luciferase expression plasmids were transfected into cells as a reference control. At 48 h, we used lysis buffer (Promega, USA) to lyse harvested cells. Firefly and Renilla luciferase activities were detected by the Dual-Luciferase Reporter Assay System (Promega, USA), according to the manufacturer's instructions.

### Tumor xenografts in mice

From the Animal Model Institute of Nanjing University (Nanjing, China), we obtained 4-week-old female BALB/c nude mice. All experimental animals were treated in accordance with the NMU Institutional Animal Care and Use Committee. For tumor growth assays, 30 female nude mice were randomly assigned to six groups and Huh-7-NC, Huh-7-pre-miR-1254, and Huh-7-miR-1254-inhibitor, Hep3B-NC, Hep3B-pre-miR-1254, Hep3B-miR-1254 stable cells (1 × 10^6^) in 100 μL PBS were injected subcutaneously into nude mice. Tumor volume was measured with Vernier calipers every 4 days and calculated using the formula: volume = (length × width^2^)/2. For tumor metastasis assays, nude mice and cell types were as above. Through tail veins, 1 × 10^6^ cells in PBS was injected into nude mice. Occurrence of metastases was imaged by an IVIS Imaging System (Caliper Life Sciences, Waltham, MA, USA). Mice were observed for four weeks and then euthanized.

### Statistical analysis

All statistical analyses used SPSS v22.0 (SPSS Inc., Chicago, USA). Each experiment was repeated three times, and experimental data are shown as mean ± standard deviation (SD). The association of miR-1254 expression with clinical and pathological features was analyzed using chi-square tests. Pearson's correlation analysis was used to determine correlations. Differences between two groups were compared using independent *t*-tests. Differences among more than two groups were compared using analysis of variance (ANOVA). *P*-values <0.05 were considered statistically significant.

## Results

### MiR-1254 is upregulated in HCC tissues and cell lines

**Figure 1:**
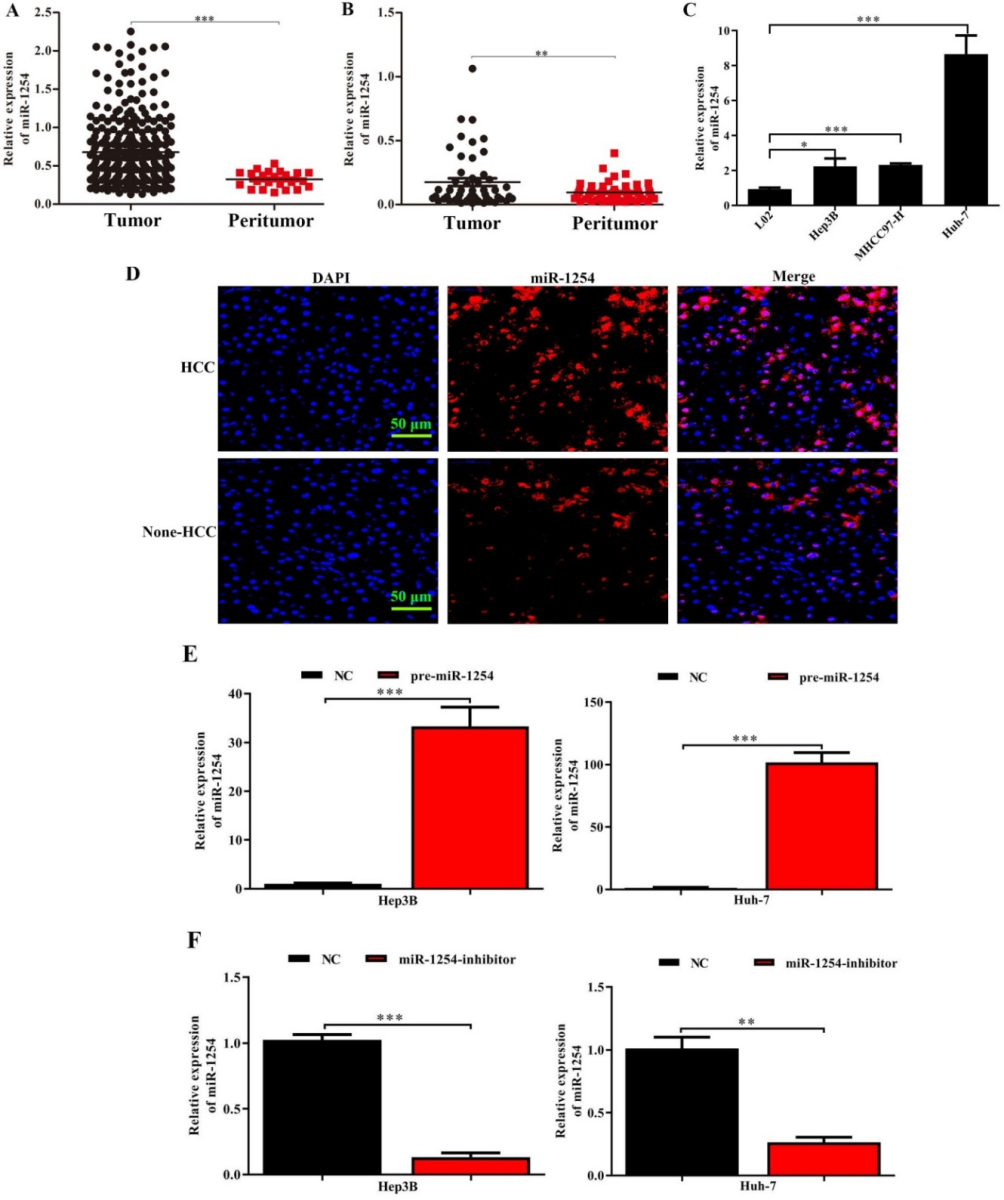
** MiR-1254 is upregulated in HCC cell lines and tissues. (A)** MiR-1254 expression in paired human HCC and adjacent normal tissues from The Cancer Genome Atlas (TCGA) database. **(B)** MiR-1254 expression in 50 paired human HCC and adjacent normal tissues from patients with HCC. **(C)** MiR-1254 expression in HCC cell lines and normal LO2 cells. **(D)** Expression of miR-1254 in HCC and adjacent normal tissues detected by fluorescence *in situ* hybridization (FISH) assay (scale bars: 50 µm). **(E and F)** Lentivirus overexpressing miR-1254 (pre-miR-1254) or with short hairpin RNA targeting miR-1254 (miR-1254-inhibitor) were used to transfect Hep3B and Huh-7 cells. Cells with empty lentiviral vectors were negative controls (NC). RT-qPCR was used to analyze expression of miR-1254 after transfection; All experiments were performed three times and data are mean ± SD. *P<0.05, **P<0.01, ***P<0.001.

Through analysis of data in The Cancer Genome Atlas (TCGA) database, we found that miR-1254 was overexpressed in HCC tissues compared with peritumor tissues (Fig. 1A). For further study, we carried out RT-qPCR to measure miR-1254 expression in 50 paired HCC tissues and adjacent normal tissues. Compared to adjacent normal samples, miR-1254 expression was significantly upregulated in HCC tissues (Fig. 1B). To explore the effect of miR-1254 on clinical and pathological features of HCC, HCC patients were divided into high and low groups based on median miR-1254 value. High levels of miR-1254 significantly linked to larger tumor size (*P*=0.019), more aggressive vascular invasion (*P*=0.033), and higher Edmondson grade (*P*=0.032) (Table 1). Expression of miR-1254 was significantly increased in HCC cell lines (Fig. 1C) compared with expression in the normal liver cell line LO2. Next, paired HCC and adjacent normal tissues were used for FISH analysis of miR-1254 expression, which further showed higher expression of miR-1254 in HCC tissues (Fig. 1D). To further investigate the effect of miR-1254 on HCC cell biological behavior, we constructed Hep3B and Huh-7 cells with stable overexpression and low expression of miR-1254. RT-qPCR results confirmed that expression of miR-1254 in HCC cells transfected with pre-miR-1254 was significantly higher than the negative control (Fig. 1E). In contrast, expression of miR-1254 in HCC cells transfected with miR-1254-inhibitor was significantly lower than the negative control (Fig. 1F).

### MiR-1254 promotes HCC cell proliferation *in vitro*

**Figure 2:**
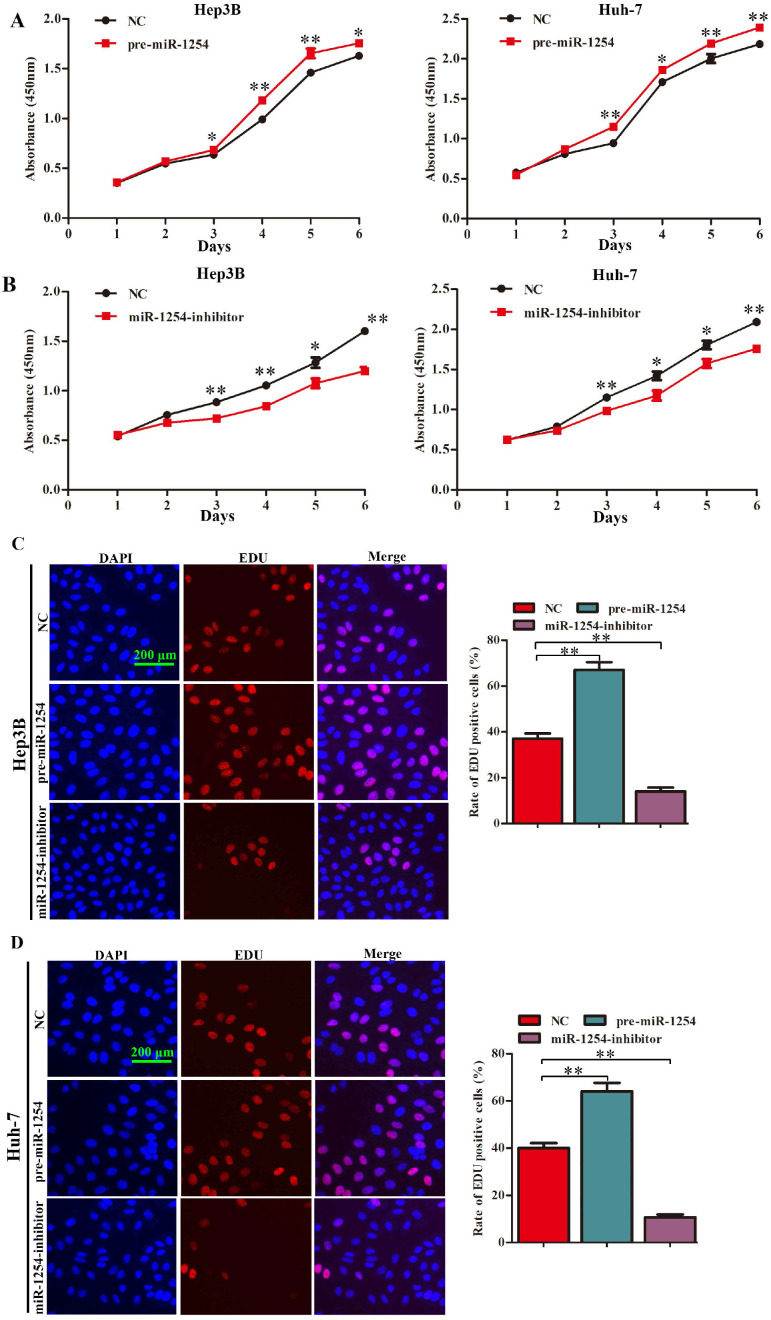

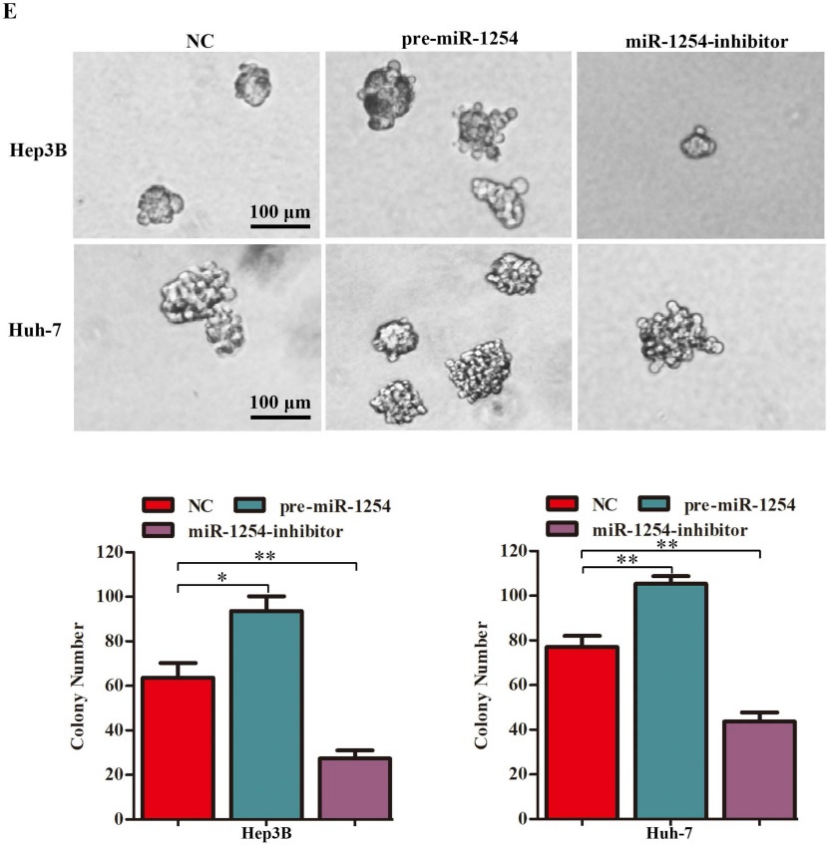
**MiR-1254 promotes HCC cell proliferation *in vitro*. (A and B**) CCK-8 assays were used to observe proliferation of overexpressed or knockdown miR-1254 in Hep3B and Huh-7 cells. **(C and D)** 5-Ethynyl-2′-deoxyuridine (EdU) proliferation assays were used to explore proliferation of Hep3B and Huh-7 cells with overexpressed or knocked down miR-1254. (red: EdU-positive; blue: DAPI; scale bars: 200 µm). **(E)** Soft agar growth assays showed growth of Hep3B and Huh-7 cells with overexpressed or knocked down miR-1254. Three independent experiments were performed for each group. All data are mean ± SD. *P<0.05, **P<0.01.

To explore the function of miR-1254 in proliferation of HCC cell lines, we conducted CCK-8, EdU, and soft agar growth assays. CCK-8 assays showed that upregulation of miR-1254 significantly promoted proliferation of Hep3B and Huh-7 cells. However, downregulation of miR-1254 significantly inhibited proliferation of the HCC cells (Fig. 2A and B). Consistent with CCK-8 assays, EdU assays showed similar effects of miR-1254 on proliferation of HCC cells (Fig. 2C and D). The capacity of cells to form clones might indicate the potential for tumor growth. In soft agar growth assays, overexpression of miR-1254 significantly promoted anchorage-independent cell growth of Hep3B and Huh-7 cells. Downregulation of miR-1254 inhibited the growth of the HCC cells (Fig. 2E). Experimental results of these assays demonstrated that miR-1254 promoted HCC cell proliferation *in vitro*.

### MiR-1254 promotes HCC cell migration, invasion, and epithelial-mesenchymal transition *in vitro*

**Figure 3:**
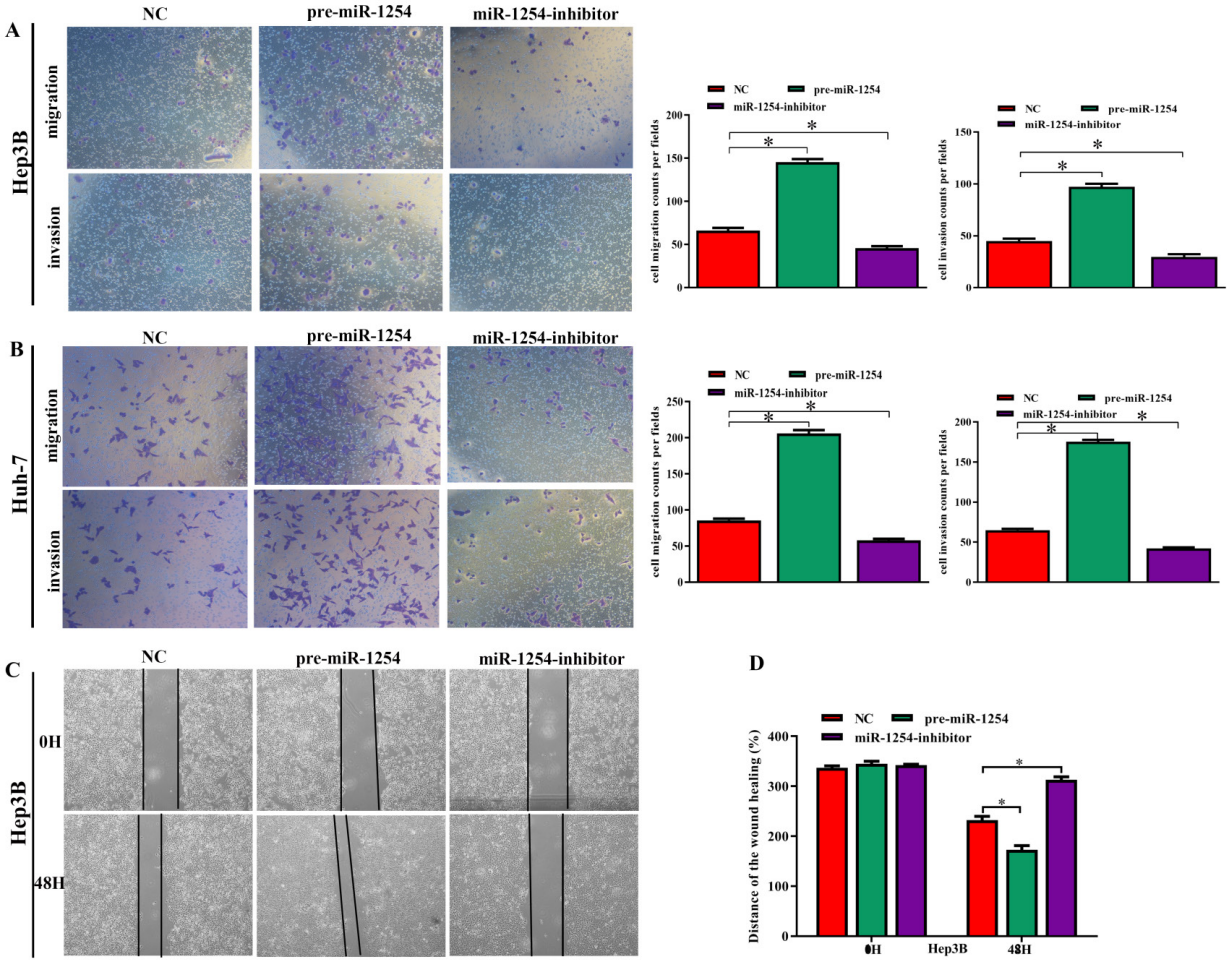

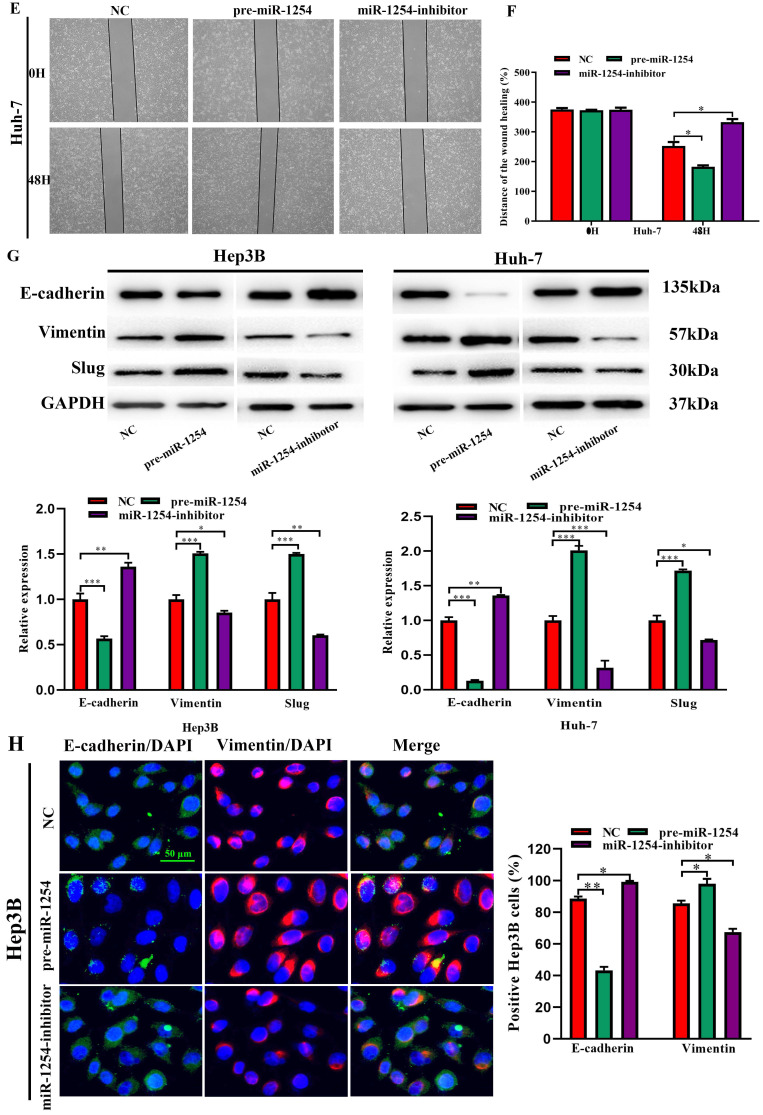

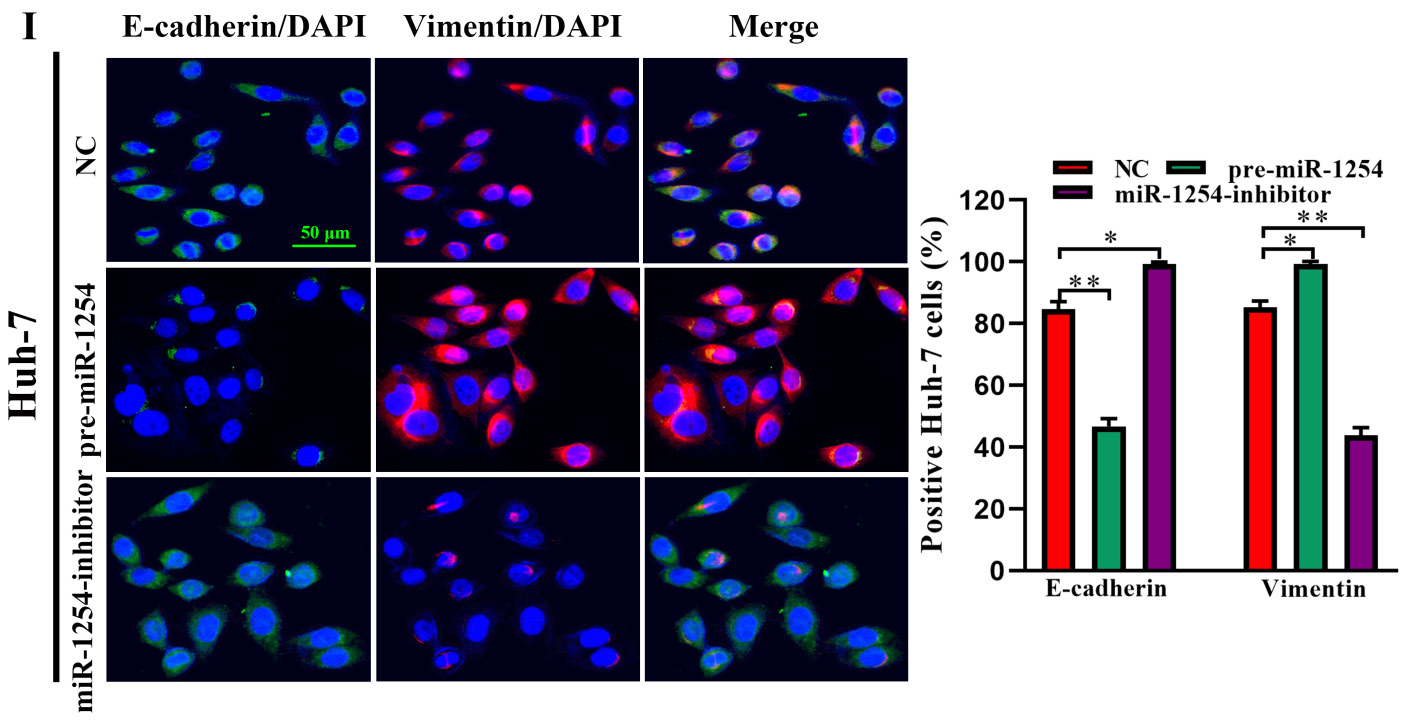
**MiR-1254 promotes HCC cell migration, invasion, and EMT *in vitro*. (A and B)** Transwell assays demonstrated migration and invasion of Hep3B and Huh-7 cells with overexpressed or knocked down miR-1254. **(C-F)** Wound-healing assays were performed in Hep3B and Huh-7 cells with overexpressed or knocked down miR-1254. **(G)** Western blots examined levels of epithelial-mesenchymal transition (EMT)-related proteins. **(H and I)** Expression of EMT markers in Hep3B and Huh-7 cells with overexpressed or knocked down miR-1254 detected by immunofluorescence (red: E-cadherin, green: vimentin, blue: DAPI) (scale bars: 50 µm). Three independent experiments were performed for each group. All data are means ± SD. *P<0.05, **P<0.01, ***P<0.001.

To further investigate the influence of miR-1254 on the migration and invasion of HCC cells *in vitro*, we used cell migration, matrigel invasion, and wound-healing assays. Transwell assays showed that overexpression of miR-1254 enhanced the migration and invasion of Hep3B and Huh-7 cells compared with control cells. In contrast, knockdown of miR-1254 restrained migration and invasion of HCC cells compared with negative controls (Fig. 3A and B). Furthermore, compared with control cells, HCC cells with miR-1254 overexpression had enhanced migration in wound healing assays, whereas HCC cells with miR-1254 knockdown showed decreased migration compared with negative controls (Fig. 3C-F). The epithelial-mesenchymal transition (EMT) process is related to the migration of cancer cells. Therefore, we examined expression levels of E-cadherin, vimentin and slug in HCC cells. Western blots showed reduced E-cadherin expression but increased vimentin and slug in Hep3B and Huh-7 cells overexpressing miR-1254. Silencing miR-1254 showed the opposite results (Fig. 3G). Similar patterns for E-cadherin and vimentin were demonstrated by immunofluorescence (Fig. 3H and I). All data supported that miR-1254 promoted HCC cell migration, invasion, and EMT *in vitro*.

### PAX5 is a functional target of miR-1254 in HCC

**Figure 4:**
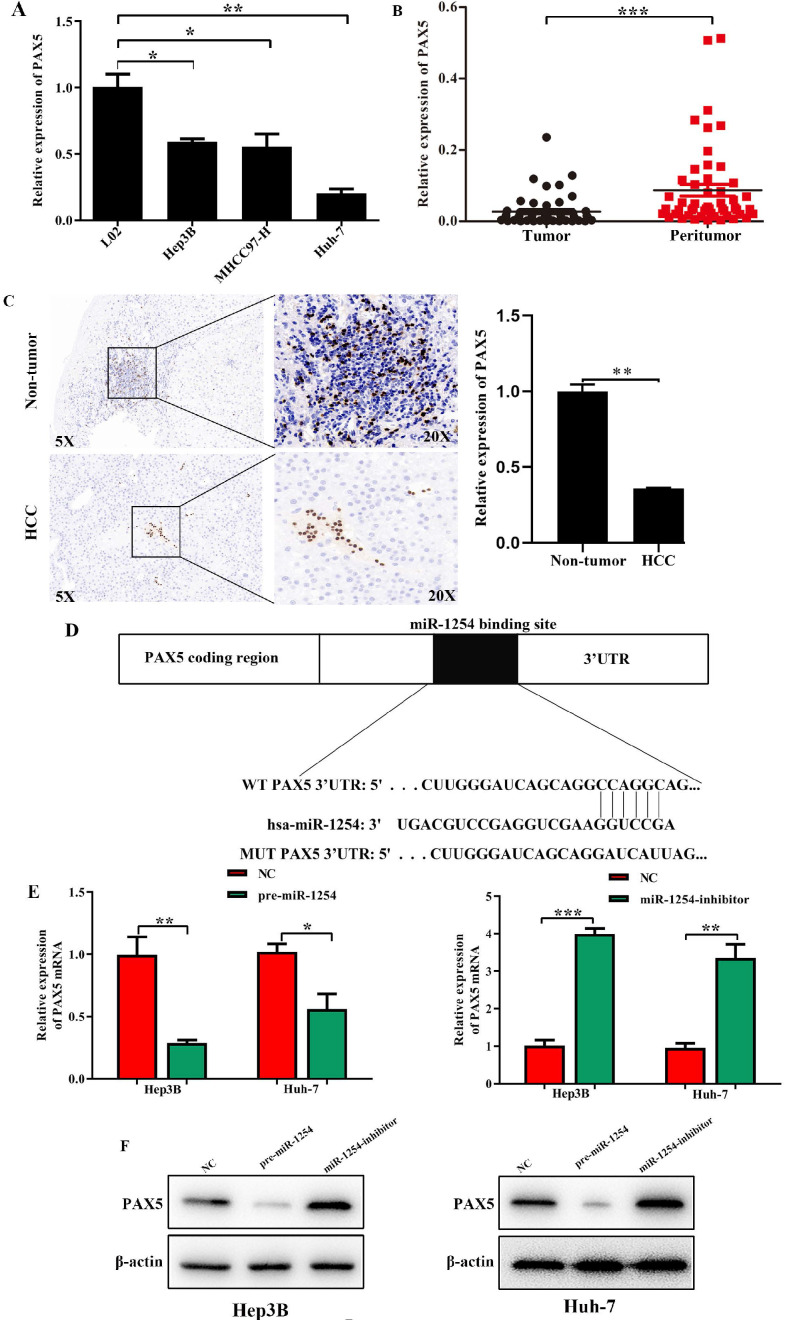

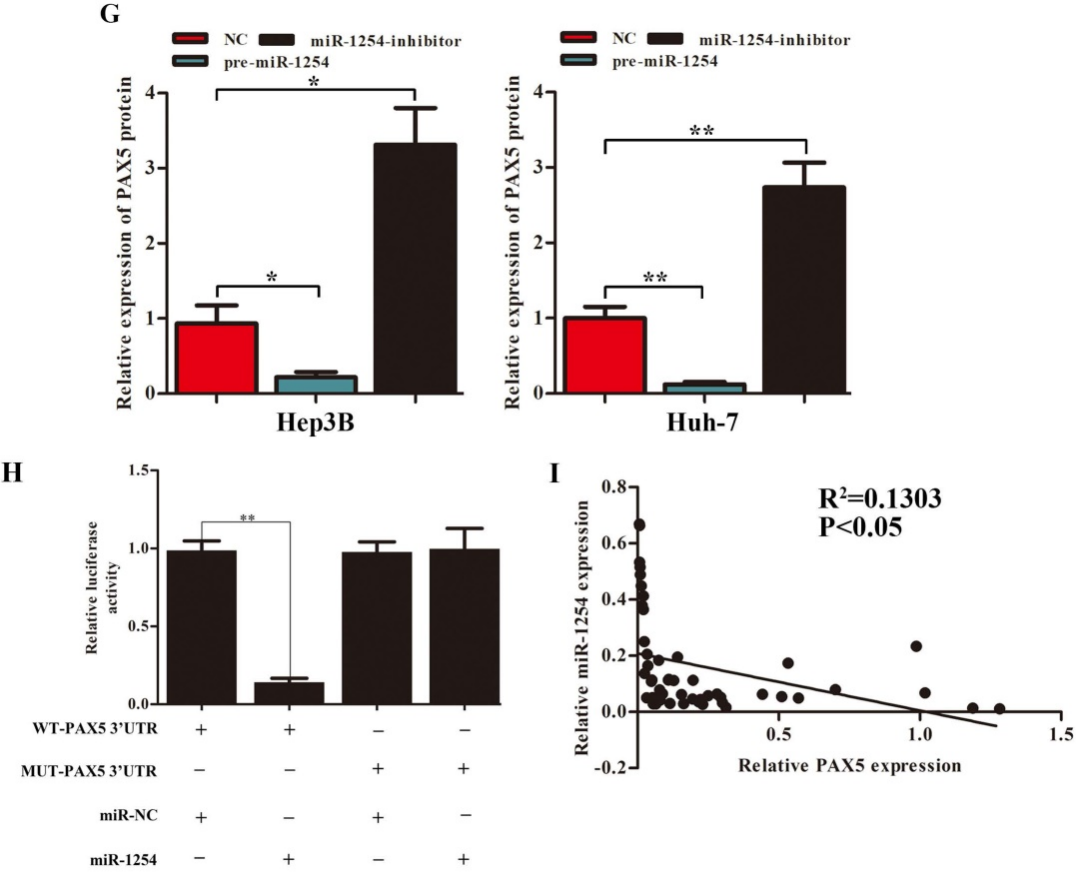
** PAX5 is a functional target of miR-1254 in HCC. (A)** RT-qPCR was used to measure mRNA of PAX5 in HCC cell lines and normal L02 cells. **(B)** RT-qPCR was used to measure mRNA of PAX5 in 50 paired human HCC and adjacent normal tissues. **(C)** Immunohistochemistry (IHC) detected expression of PAX5 in HCC and adjacent non-tumor tissues. **(D)** Target Scan was used to predict potential miR-1254 binding sites in wild-type (WT) PAX5 3'-UTR or mutant-type (MUT) PAX5 3'-UTR. **(E)** mRNA levels of PAX5 in over-expressed miR-1254 Hep3B and Huh-7 cells with overexpressed or knocked down miR-1254. Cells transfected with empty lentiviral vectors were negative controls (NC). **(F and G)** PAX5 protein in Hep3B and Huh-7 cells with with overexpressed or knocked down miR-1254. **(H)** Dual-luciferase reporter assays to investigate effects of miR-1254 expression on activities of WT and MUT PAX5 3'-UTR. **(I)** Negative correlation between the levels of miR-1254 and PAX5 expression in HCC tissues. All data are means ± SD. *P<0.05, **P<0.01, ***P<0.001.

We employed TargetScan Software (version 7.1; http://www.targetscan.org/vert_71) to screen for targeted mRNA of miR-1254. Among the screened target candidates, we chose PAX5 as a potential target. We used RT-qPCR to investigate the expression of PAX5 mRNA in HCC tissues, paired normal tissues, the normal hepatic cell line LO2, and HCC lines. Compared with expression in LO2 cells, expression of PAX5 mRNA was lower in HCC lines (Fig. 4A). Similarly, PAX5 mRNA expression was lower in HCC tissues compared to peritumor tissues (Fig. 4B). Lower PAX5 expression was detected in tumor tissues than in peritumor tissues by IHC analysis (Fig. 4C). We predicted that the seed region of miR-1254 bound to the PAX5 wild-type (WT) 3'-UTR directly, but not the PAX5 mutant-type (MUT) 3'-UTR (Fig. 4D). RT-qPCR showed the effect of miR-1254 on expression of PAX5 in Hep3B and Huh-7 cells. Overexpression of miR-1254 decreased expression of PAX5, but silencing miR-1254 gave the opposite result (Fig. 4E). Similar results were seen on western blots (Fig. 4F and G). Overexpression of miR-1254 reduced the luciferase activity of only WT-3′-UTR of PAX5, not the MUT-3'-UTR of PAX5 (Fig. 4H). RT-qPCR was applied to study the expression of miR-1254 and PAX5 mRNA in HCC and normal tissues. Overexpression of miR-1254 was correlated with reduced expression of PAX5 (R^2^=0.1303, P<0.05) (Fig. 4I). These results indicated that PAX5 was a functional target of miR-1254 in HCC.

### Restoration of PAX5 reverses miR-1254 effects on proliferation, migration, and invasion of HCC cells

**Figure 5:**
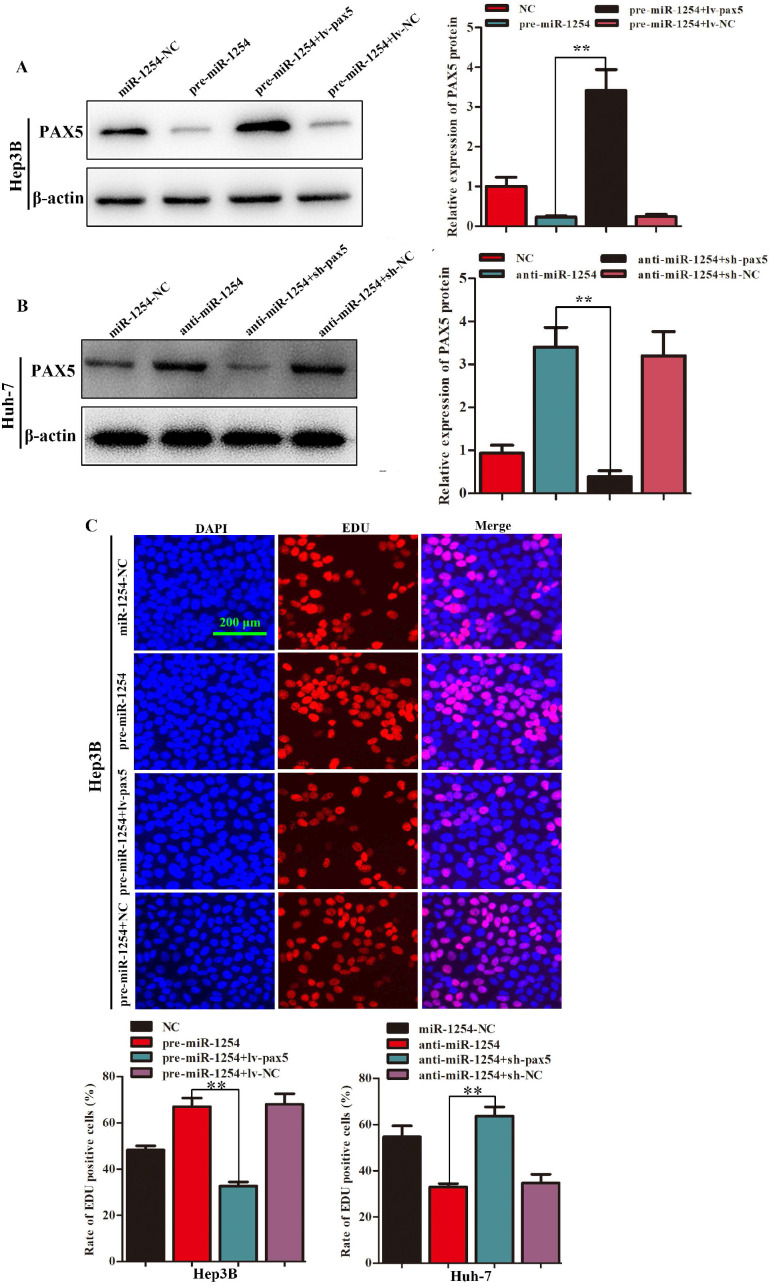

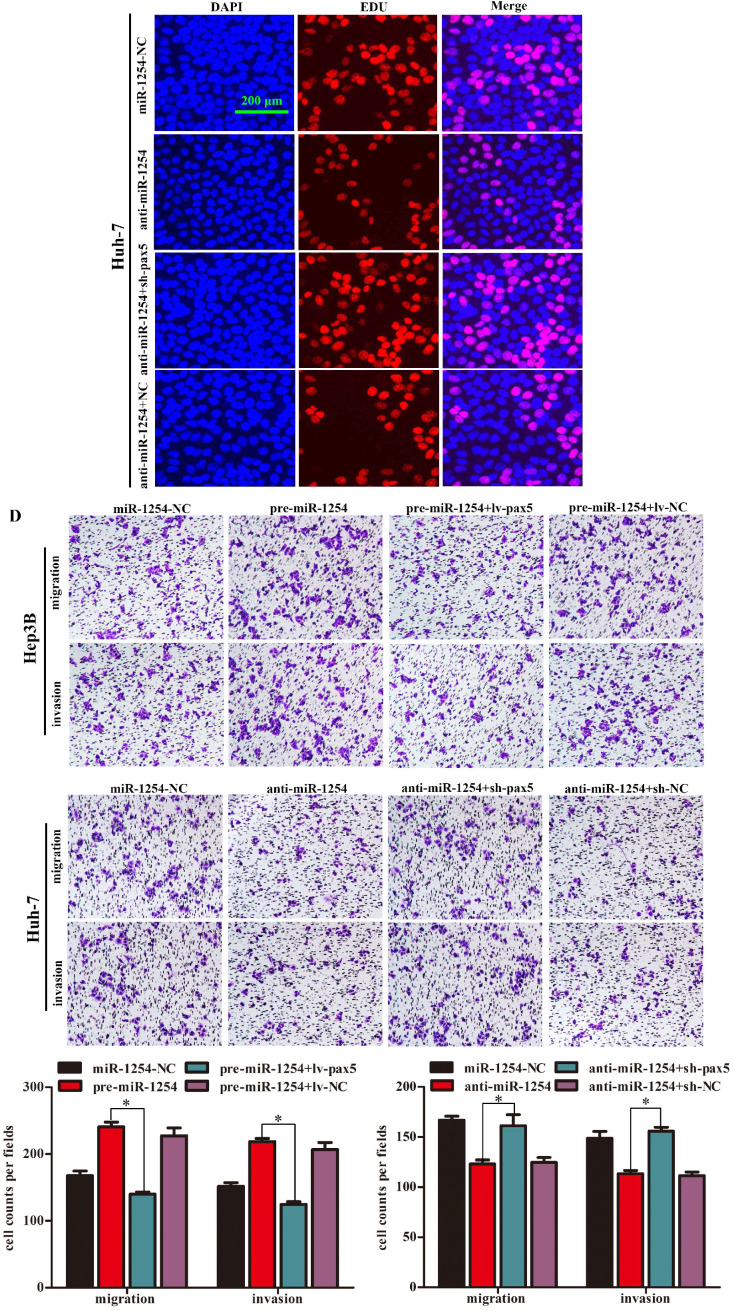
**Restoration of PAX5 reverses effects of miR-1254 on migration, invasion, and proliferation of HCC cells. (A)** Western blots of PAX5 protein in Hep3B cells transfected with overexpressing miR-1254 lentivirus (pre-miR-1254) or overexpressing PAX5 lentivirus (lv-PAX5). Cells transfected with empty lentiviral vectors were the negative controls. **(B)** Western blots for PAX5 protein in Huh-7 cells transfected with miR-1254 knockdown lentivirus (anti-miR-1254) or PAX5 lentivirus knockdown (sh-PAX5). **(C)** EdU proliferation assays of Hep3B cells transfected with pre-miR-1254 or lv-PAX5, and Huh-7 cells transfected with anti-miR-1254 or sh-PAX5. **(D)** Transwell assays of Hep3B cells transfected with pre-miR-1254 or lv-PAX5, and Huh-7 cells transfected with anti-miR-1254 or sh-PAX5. Three independent experiments were performed for each group. All data are means ± SD. *P<0.05, **P<0.01.

To further determine the influence of miR-1254 on proliferation, migration, and invasion of HCC through PAX5, we transfected Hep3B cells using pre-miR-1254, pre-miR-1254+lv-PAX5 lentiviruses and the respective controls. Huh-7 cells were transfected with anti-miR-1254, anti-miR-1254+sh-PAX5 lentiviruses and the respective controls. Western blots confirmed that overexpressed miR-1254 decreased PAX5 protein levels, while overexpressed PAX5 reversed the inhibitory effect on PAX5 protein levels in Hep3B cells (Fig. 5A). Knockdown of miR-1254 increased PAX5 protein levels, while knockdown of PAX5 reversed the enhanced effect on PAX5 protein levels in Huh-7 cells (Fig. 5B). In EdU assays, overexpressed miR-1254 promoted proliferation of Hep3B cells, and overexpressed PAX5 attenuated the promotion. Knockdown of miR-1254 inhibited proliferation of Huh-7 cells, and knockdown of PAX5 reversed the inhibition of proliferation (Fig. 5C). In migration and invasion assays, Hep3B cells transfected with pre-miR-1254+lv-PAX5 lentiviruses showed decreased migration and invasion compared with cells transfected with pre-miR-1254. Huh-7 cells transfected with anti-miR-1254+sh-PAX5 showed enhanced migration and invasion compared with cells transfected anti-miR-1254 (Fig. 5D). According to these results, restoration of PAX5 reversed the effects of miR-1254 on proliferation, migration, and invasion of HCC cells.

### MiR-1254 regulates the YAP-Hippo signaling pathway through PAX5

**Figure 6:**
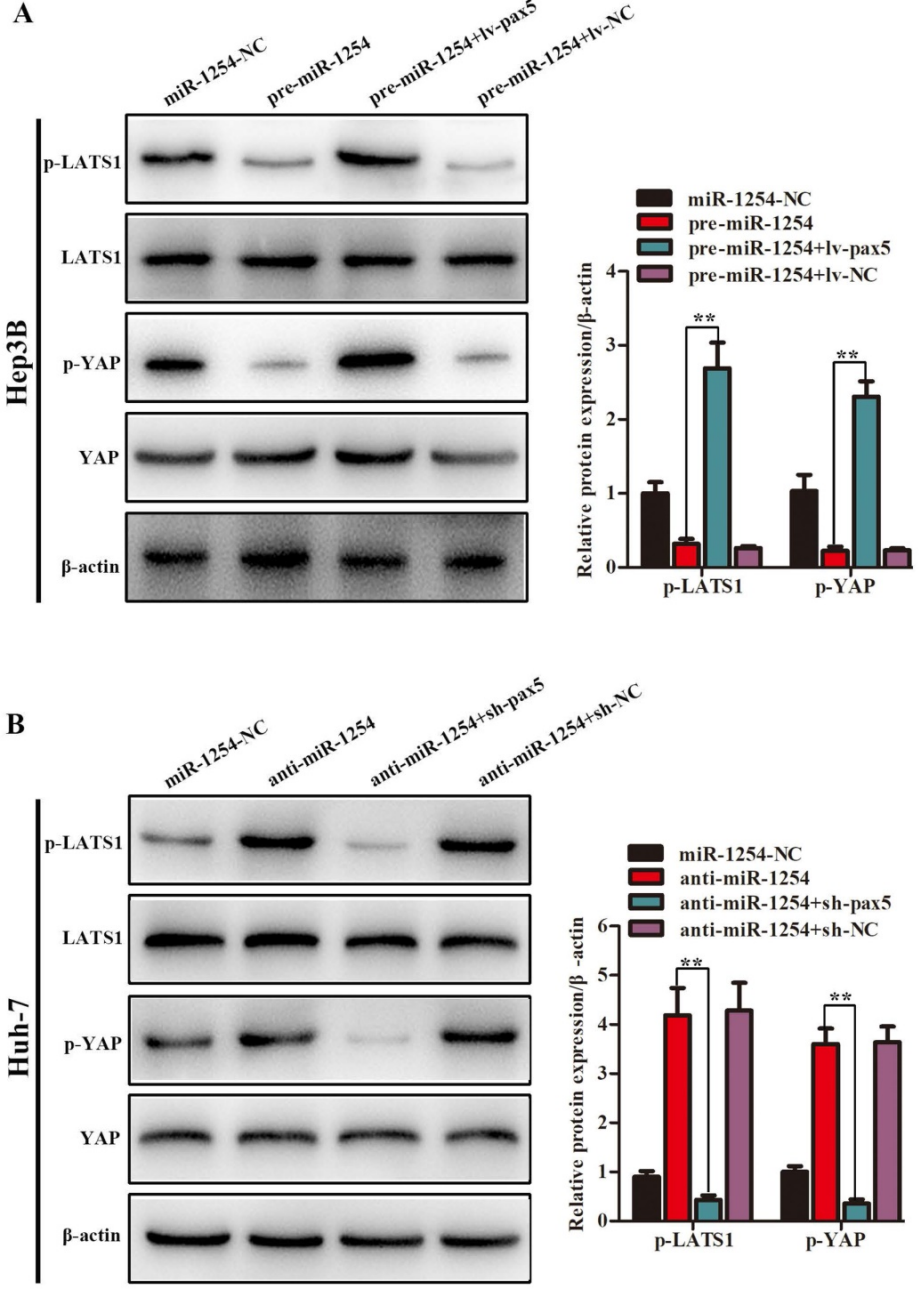

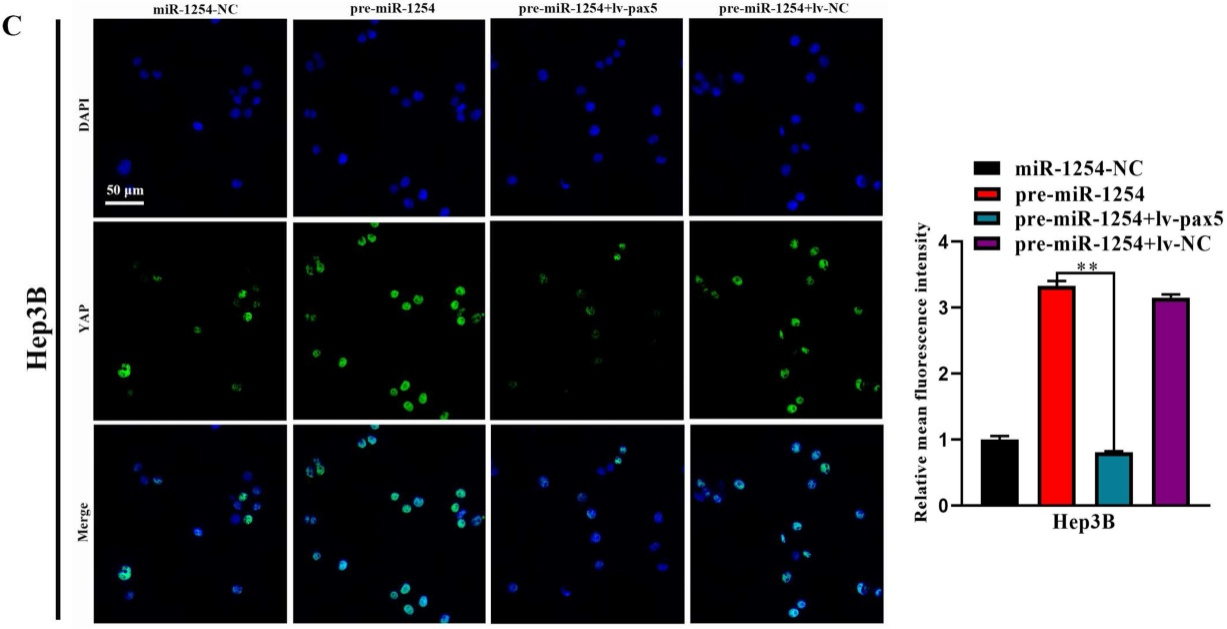

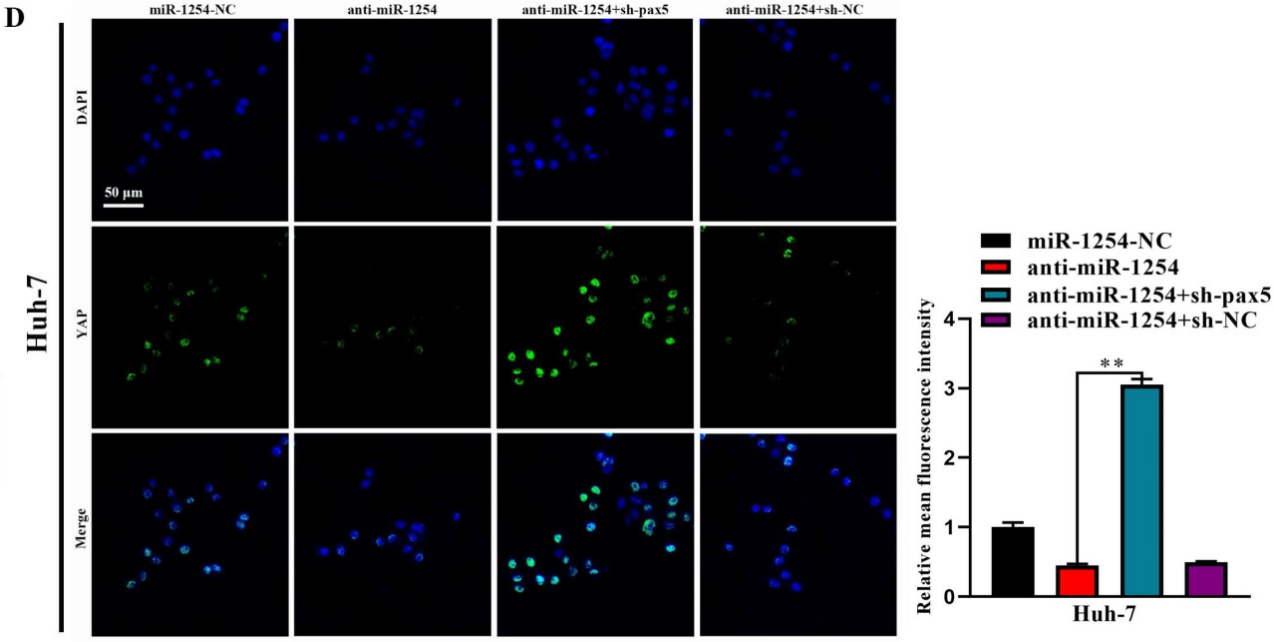
** MiR-1254 regulates the YAP-Hippo signaling pathway through PAX5. (A)** Western blots of effects of overexpressing miR-1254 or PAX5 on protein levels of p-LATS1 and p-YAP in Hep3B cells.** (B)** Western blots of effects of knockdown of miR-1254 or PAX5 on protein levels of p-LATS1 and p-YAP in Huh-7 cells. **(C)** Immunofluorescence showed nuclear and cytoplasmic retention of YAP with overexpressed miR-1254 or PAX5 in Hep3B cells. **(D)** Immunofluorescence showed nuclear and cytoplasmic retention of YAP with knockdown of miR-1254 or PAX5 in Huh-7 cells. (scale bars: 20 µm). All data are means ± SD. **P<0.01.

In our study, we found that the Hippo signaling pathway might be the underlying mechanism of the miR-1254/PAX5 axis that promoted HCC progression. Western blots showed that expression of phosphorylated-LATS1 (p-LATS1) and phosphorylated-YAP (p-YAP) were decreased in miR-1254 overexpressing Hep3B cells. In contrast, overexpression of PAX5 reversed the inhibition of p-LATS1 and p-YAP protein levels in miR-1254 overexpressing Hep3B cells (Fig. 6A). We found that levels of p-LATS1 and p-YAP were increased in miR-1254 knockdown Huh-7 cells. Knockdown of PAX5 reversed the promotion of p-LATS1 and p-YAP protein levels in miR-1254 knockdown Huh-7 cells (Fig. 6B). Similarly, immunofluorescence showed that overexpressing miR-1254 led to retaining more YAP in nuclei, and promoted transcriptional activity in Hep3B cells. Overexpressed PAX5 significantly reduced nuclear retention of YAP caused by miR-1254 (Fig. 6C). Silencing miR-1254 increased the cytoplasmic sequestration of YAP in Huh-7 cells. Downregulation of PAX5 reversed this result in Huh-7 cells (Fig. 6D). These results supported that miR-1254 regulated the YAP-Hippo signaling pathway through PAX5.

### MiR-1254 promotes xenograft tumor growth *in vivo*

**Figure 7:**
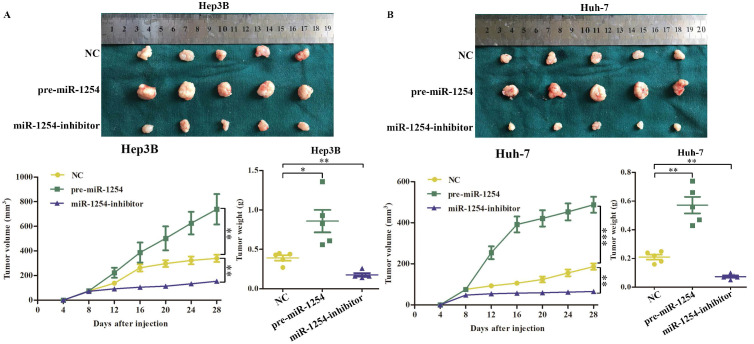

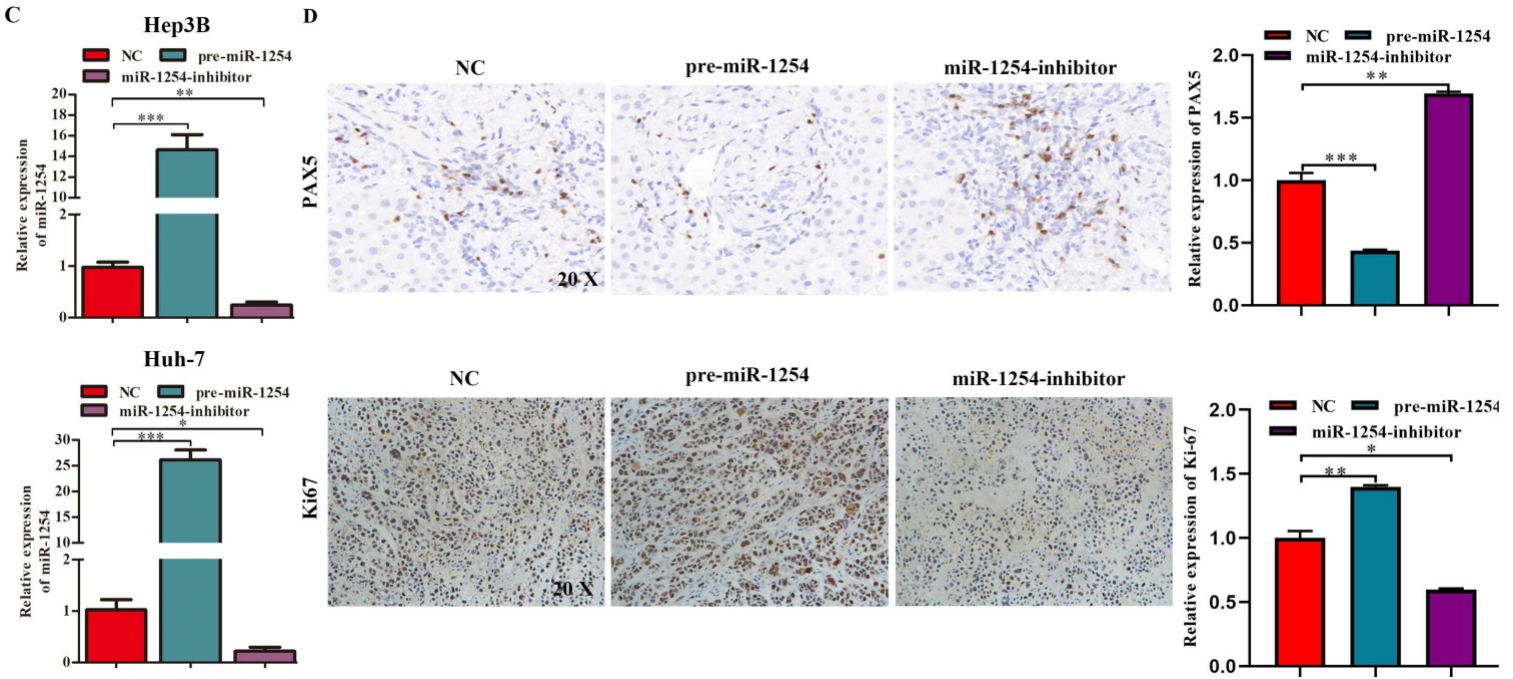
**MiR-1254 promotes xenograft tumor growth *in vivo*. (A and B)** Upper: HCC tumors from mice inoculated with Hep3B cells or Huh-7 cells transfected with empty lentiviral vectors (NC), pre-miR-1254, or miR-1254-inhibitor lentiviruses; Lower: growth curves of tumor volume and weight in mice injected with transfected Hep3B cells or Huh-7 cells. **(C)** Relative expression of miR-1254 in the above tumors from the mice measured by RT-qPCR.** (D)** Expression of PAX5 and Ki67 detected by immunohistochemistry (IHC) of tumor tissues from mice inoculated with Hep3B cells or Huh-7 cells. All data are means ± SD. *P<0.05, **P<0.01, ***P<0.001.

To determine the effects of miR-1254 on tumor growth *in vivo*, we transfected Hep3B cells and Huh-7 cells with miR-1254 overexpressing or knockdown lentiviruses, then injected these cells into nude mice subcutaneously. All mice were observed for 28 days, and tumors were examined every four days. Overexpression of miR-1254 increased volume and weight of tumors from HCC cells, and knockdown of miR-1254 inhibited increasement of volume and weight of tumors (Fig. 7A and B). The relative expression of miR-1254 in tumor tissues was confirmed by RT-qPCR. RT-qPCR detected higher levels of miR-1254 in tumors of Hep3B and Huh-7 cells with overexpressed miR-1254 than in tumors derived from knockdown miR-1254 HCC cells (Fig. 7C). Expression of PAX5 and Ki-67 in tumor tissues was detected by immunohistochemistry. Overexpression of miR-1254 in tumor tissues led to less expression of PAX5 but more of Ki-67, while knockdown of miR-1254 led to the reverse result compared with controls (Fig. 7D). These results confirmed that miR-1254 promoted xenograft tumor growth *in vivo*.

### MiR-1254 promotes HCC metastasis *in vivo*

**Figure 8:**
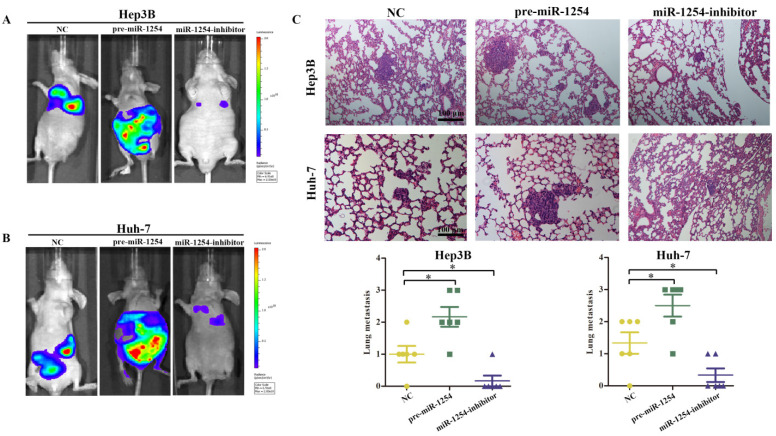

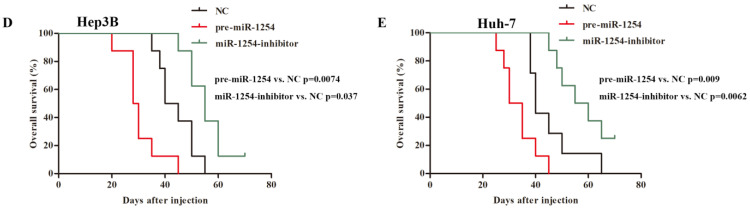
**MiR-1254 promotes HCC metastasis *in vivo*. (A and B)** Bioluminescent images of mice injected with Hep3B cells or Huh-7 cells transfected with empty lentiviral vectors (NC), pre-miR-1254, or miR-1254-inhibitor lentiviruses through tail veins 6 weeks later. **(C)** Metastatic nodules in lungs of mice injected with transfected Hep3B cells or Huh-7 cells through tail veins. **(D and E)** Overall survival of groups of mice injected with transfected Hep3B cells or Huh-7 cells. All data are means ± SD. *P<0.05.

To explore the effect of miR-1254 on HCC metastasis, we injected the stably transfected HCC cells into nude mice via tail vein. Mice were monitored by bioluminescence imaging, which showed that groups injected with cells overexpressing miR-1254 had more abdominal metastases, and the knockdown group had less abdominal metastases compared with the control group (Fig. 8A and B). Similarly, clinical pathology showed that more metastatic nodules were seen in lung tissues of mice injected with HCC cells overexpressing miR-1254 compared with controls. In contrast, knockdown miR-1254 HCC cells caused fewer lung metastatic nodules (Fig. 8C). After monitoring for about 70 days, survival outcomes of the three groups were determined. The miR-1254 overexpression group had worse survival than the control group, while the miR-1254 knockdown group had better survival than the control group (Fig. 8D and E). All data supported that miR-1254 promoted HCC metastasis *in vivo*.

## Discussion

MiRNAs are reported to have an important role in tumorigenesis as carcinogenic factors or tumor suppressor factors by targeting mRNAs 29. Previous studies showed that aberrant expression of miRNAs promoted proliferation, migration, invasion and metastasis of HCC cells 30. It was reported that miR-1254 had anti-tumor activity in oral squamous cell carcinoma by targeting CD36 31. Another study found that miR-1254 targeted secreted frizzled related protein 1 (SFRP1), a Wnt/β-catenin pathway antagonist, to promote lung cancer cell proliferation 32. However, effects of miR-1254 on the biological function of HCC cells were unclear. In this study, we demonstrated that miR-1254 was overexpressed in HCC tissues and cell lines, and miR-1254 were linked to larger tumor size, more aggressive vascular invasion and higher Edmondson grade, indicating that miR-1254 might be a potential therapeutic target for HCC.

Many cancer cells of epithelial origin can metastasize with the loss of epithelial characteristics and the acquisition of mesenchymal phenotypes. This phenomenon is called the epithelial-mesenchymal transition (EMT) and is important in malignant tumor metastasis 33. Usually, increased expression of vimentin and slug and decreased expression of E-cadherin can be detected in EMT 34. We detected these changes with western blots and immunofluorescence in miR-1254-overexpressing HCC cells, meaning EMT was enhanced in these HCC cells. In addition, we demonstrated that miR-1254 promoted the proliferation, migration and invasion of HCC cells, which was confirmed by gain-of-function and loss-of-function experiments *in vitro*. These findings also supported that miR-1254 promoted migration and invasion of HCC cells.

As we described, miRNAs usually target 3′-UTRs of downstream mRNAs to regulate progression of tumor cells. To further study the mechanism of miR-1254 in biological effects on HCC cells, we predicted potential targets of miR-1254 using bioinformatic website tools. PAX5 was reported to act as a tumor suppressor in HCC 35, so we were interested in PAX5 among the screened candidates. It was found that PAX5 inhibited breast cancer proliferation and migration through upregulating miR-215 and was regarded as a tumor suppressor 17. In addition, another study reported that PAX5 suppressed miR-155 in breast cancer cells and suppressed PAX5, forming a feedback loop 36. In our research, we confirmed that miR-1254 promoted progression of HCC *in vitro* and *in vivo* by directly targeting PAX5, based on the following evidence: (1) Lower expression of PAX5 was seen in HCC tissues and cell lines at the mRNA level, indicating that PAX5 could be a tumor suppressor in HCC cells. (2) Dual luciferase reporter assays showed that miR-1254 had an effect on the luciferase activity of WT PAX5 3′-UTR but not MUT PAX5 3′-UTR, suggesting that PAX5 was the direct target of miR-1254. (3) Overexpression of miR-1254 decreased levels of PAX5 mRNA in HCC cells. (4) Migration and invasion assays confirmed restoration of PAX5 reversed the effect of overexpression of miR-1254 in HCC cells. EdU assays were also consistent with these results. (5) Tumor xenografts in mice supported that miR-1254 promoted tumor growth and metastasis. (6) Compared with a control group, mice treated with miR-1254-overexpressing HCC cells had poor survival.

Dysregulation of signaling pathways is involved in carcinogenesis. It was reported that PAX5 had anti-tumor ability in liver carcinogenesis through directly regulating the p53 signaling pathway 35. In this study, we found that miR-1254 inhibited Hippo pathway activity through PAX5. We found that overexpression of miR-1254 decreased levels of p-LATS1 and p-YAP, and downregulation of miR-1254 had the opposite result. Moreover, we confirmed that upregulation or downregulation of PAX5 reversed the effects on the Hippo signaling pathway of, respectively, overexpressing or downregulating miR-1254. However, we detected only variations of LAST1, p-LAST1, YAP, and p-YAP with western blots. Other components in the Hippo signaling pathway were not investigated. Thus, more experiments are required to improve understanding of the specific mechanism of PAX5 on the Hippo signaling pathway. All these studies indicate that the anti-tumor activity of PAX5 in HCC may involve more mechanisms, to be studied in the future.

In summary, our study demonstrated that miR-1254 was upregulated in HCC tissues and cell lines. High expression of miR-1254 was linked to larger tumor size, more aggressive vascular invasion and higher Edmondson grade in HCC patients. Moreover, overexpressing miR-1254 enhanced proliferation, migration and invasion of HCC cells *in vitro* and promoted xenograft tumor growth and metastasis *in vivo*. Furthermore, we confirmed that miR-1254 inhibited Hippo signaling activity by directly binding to the 3′-UTR of PAX5 to suppress its expression. Thus, our findings suggest that miR-1254 could be a potential therapeutic target for HCC.

## Figures and Tables

**Table 1:** Association between miR-1254 expression levels and clinicopathologic features in patients with hepatocellular carcinoma

Characteristics	Number (n=50)	miR-1254 expression levels		*P*-value
		Low (n=19)	High (n=31)	
**Age (years)**				0.409
≤60	15	7	8	
>60	35	12	23	
**Gender**				0.198
Male	37	16	21	
Female	13	3	10	
**HBV infection**				0.326
Negative	12	6	6	
Positive	38	13	25	
**Liver cirrhosis**				0.261
Absent	7	4	3	
Present	43	15	28	
**AFP (ng/ml)**				0.082
≤20	14	8	6	
>20	36	11	25	
**Tumor size (cm)**				0.019*
≤5	29	15	14	
>5	21	4	17	
**Tumor multiplicity**				0.392
Single	36	15	21	
Multiple	14	4	10	
**Vascular invasion**				0.033*
No	33	16	17	
Yes	17	3	14	
**Edmondson grade**				0.032*
I+II	30	15	15	
III+IV	20	4	16	

HBV: hepatitis B virus AFP: alpha-fetoprotein; *p < 0.05, statistically significant difference.

## References

[1] Bray F, Ferlay J, Soerjomataram I (2018). Global cancer statistics 2018: GLOBOCAN estimates of incidence and mortality worldwide for 36 cancers in 185 countries. CA Cancer J Clin.

[2] Feng RM, Zong YN, Cao SM (2019). Current cancer situation in China: good or bad news from the 2018 Global Cancer Statistics?. Cancer Commun (Lond).

[3] Galle PR, Tovoli F, Foerster F (2017). The treatment of intermediate stage tumours beyond TACE: From surgery to systemic therapy. J Hepatol.

[4] Treiber T, Treiber N, Meister G (2019). Regulation of microRNA biogenesis and its crosstalk with other cellular pathways. Nat Rev Mol Cell Biol.

[5] Lytle JR, Yario TA, Steitz JA (2007). Target mRNAs are repressed as efficiently by microRNA-binding sites in the 5' UTR as in the 3' UTR. Proc Natl Acad Sci U S A.

[6] Orom UA, Nielsen FC, Lund AH (2008). MicroRNA-10a binds the 5'UTR of ribosomal protein mRNAs and enhances their translation. Mol Cell.

[7] Ruksha TG (2019). MicroRNAs' control of cancer cell dormancy. Cell Div.

[8] Pu M, Li C, Qi X (2017). MiR-1254 suppresses HO-1 expression through seed region-dependent silencing and non-seed interaction with TFAP2A transcript to attenuate NSCLC growth. Plos Genet.

[9] Zhou J, Liu X, Wang CH (2018). Decreased expression of miR-1254 is associated with cancer aggressiveness and predicts poor outcome in cervical cancer. Eur Rev Med Pharmacol Sci.

[10] Li B, Chen P, Wang J (2018). MicroRNA-1254 exerts oncogenic effects by directly targeting RASSF9 in human breast cancer. Int J Oncol.

[11] Jiang M, Shi L, Yang C (2019). miR-1254 inhibits cell proliferation, migration, and invasion by down-regulating Smurf1 in gastric cancer. Cell Death Dis.

[12] He D, Yue Z, Liu L (2019). Long noncoding RNA ABHD11-AS1 promote cells proliferation and invasion of colorectal cancer via regulating the miR-1254-WNT11 pathway. J Cell Physiol.

[13] Schafer BW (1998). Emerging roles for PAX transcription factors in cancer biology. Gen Physiol Biophys.

[14] Robson EJ, He SJ, Eccles MR (2006). A PANorama of PAX genes in cancer and development. Nat Rev Cancer.

[15] Gu Z, Churchman ML, Roberts KG (2019). PAX5-driven subtypes of B-progenitor acute lymphoblastic leukemia. Nat Genet.

[16] Li X, Cheung KF, Ma X (2012). Epigenetic inactivation of paired box gene 5, a novel tumor suppressor gene, through direct upregulation of p53 is associated with prognosis in gastric cancer patients. Oncogene.

[17] Leblanc N, Harquail J, Crapoulet N (2018). Pax-5 Inhibits Breast Cancer Proliferation Through MiR-215 Up-regulation. Anticancer Res.

[18] Zhao L, Li S, Gan L (2016). Paired box 5 is a frequently methylated lung cancer tumour suppressor gene interfering beta-catenin signalling and GADD45G expression. J Cell Mol Med.

[19] Taniguchi K, Karin M (2018). NF-kappaB, inflammation, immunity and cancer: coming of age. Nat Rev Immunol.

[20] Nusse R, Clevers H (2017). Wnt/beta-Catenin Signaling, Disease, and Emerging Therapeutic Modalities. Cell.

[21] Mossmann D, Park S, Hall MN (2018). mTOR signalling and cellular metabolism are mutual determinants in cancer. Nat Rev Cancer.

[22] Hoxhaj G, Manning BD (2020). The PI3K-AKT network at the interface of oncogenic signalling and cancer metabolism. Nat Rev Cancer.

[23] Yu FX, Zhao B, Guan KL (2015). Hippo Pathway in Organ Size Control, Tissue Homeostasis, and Cancer. Cell.

[24] Guo Y, Cui J, Ji Z (2017). miR-302/367/LATS2/YAP pathway is essential for prostate tumor-propagating cells and promotes the development of castration resistance. Oncogene.

[25] Guan L, Li T, Ai N (2019). MEIS2C and MEIS2D promote tumor progression via Wnt/beta-catenin and hippo/YAP signaling in hepatocellular carcinoma. J Exp Clin Cancer Res.

[26] Zheng X, Chen L, Zhou Y (2019). A novel protein encoded by a circular RNA circPPP1R12A promotes tumor pathogenesis and metastasis of colon cancer via Hippo-YAP signaling. Mol Cancer.

[27] Kang W, Huang T, Zhou Y (2018). miR-375 is involved in Hippo pathway by targeting YAP1/TEAD4-CTGF axis in gastric carcinogenesis. Cell Death Dis.

[28] Mo JS, Park HW, Guan KL (2014). The Hippo signaling pathway in stem cell biology and cancer. Embo Rep.

[29] Lin S, Gregory RI (2015). MicroRNA biogenesis pathways in cancer. Nat Rev Cancer.

[30] Callegari E, Gramantieri L, Domenicali M (2015). MicroRNAs in liver cancer: a model for investigating pathogenesis and novel therapeutic approaches. Cell Death Differ.

[31] Chen R, Zhang Y, Zhang X (2019). MiR-1254 Functions as a Tumor Suppressor in Oral Squamous Cell Carcinoma by Targeting CD36. Technol Cancer Res Treat.

[32] Li H, Yang T, Shang D (2017). miR-1254 promotes lung cancer cell proliferation by targeting SFRP1. Biomed Pharmacother.

[33] Nieto MA, Huang RY, Jackson RA (2016). EMT: 2016. Cell.

[34] Dongre A, Weinberg RA (2019). New insights into the mechanisms of epithelial-mesenchymal transition and implications for cancer. Nat Rev Mol Cell Biol.

[35] Liu W, Li X, Chu ES (2011). Paired box gene 5 is a novel tumor suppressor in hepatocellular carcinoma through interaction with p53 signaling pathway. Hepatology.

[36] Harquail J, LeBlanc N, Landry C (2018). Pax-5 Inhibits NF-kappaB Activity in Breast Cancer Cells Through IKKepsilon and miRNA-155 Effectors. J Mammary Gland Biol Neoplasia.

